# The short-term efficacy and safety of induction chemotherapy combined with PD-1 inhibitor or anti-EGFR in locoregionally advanced nasopharyngeal carcinoma

**DOI:** 10.3389/fonc.2023.1110281

**Published:** 2023-04-21

**Authors:** Xiaoyong Xiang, Peng Chen, Fengming Lan, Li Ma, Jing Jin, Ye Zhang

**Affiliations:** ^1^ Department of Radiation Oncology, National Cancer Center/National Clinical Research Center for Cancer/Cancer Hospital & Shenzhen Hospital, Chinese Academy of Medical Sciences and Peking Union Medical College, Shenzhen, China; ^2^ Department of Radiation Oncology, National Cancer Center/National Clinical Research Center for Cancer/Cancer Hospital, Chinese Academy of Medical Sciences and Peking Union Medical College, Beijing, China

**Keywords:** nasopharyngeal carcinoma, locoregionally advanced, induction chemotherapy, short-term efficacy, PD-1

## Abstract

**Purpose:**

This study aimed to investigate the short-term efficacy and safety of induction chemotherapy (IC) combined with PD-1 inhibitor or anti-EGFR in the treatment of locoregionally advanced nasopharyngeal carcinoma (LA-NPC).

**Methods and materials:**

We retrospectively reviewed the clinical data of 206 patients with LA-NPC, including IC combined with anti-PD-1 (57 patients), IC combined with anti-EGFR (28 patients), and IC alone (121 patients). The short-term efficacy was assessed at the end of IC and one month after overall treatment. According to the RECIST v1.1, the short-term efficacy of cervical lymph nodes and primary nasopharynx foci was divided into complete remission (CR), partial remission (PR), stable disease (SD), and progressive disease (PD). The overall response (ORR) was defined as the sum of CR and PR. Acute toxicities were graded according to the CTCAE v5.0. One-way analysis of variance (ANOVA) was used to compare differences in the numerical variables among groups. Fisher Freeman-Halton test or Pearson Chi-square test was used to compare classified variables.

**Results:**

The ORR rates of primary nasopharynx foci in IC, anti-EGFR, and anti-PD-1 group were 68.60%, 67.9%, and 94.7%, respectively, and the corresponding rates of ORR in cervical lymph nodes were 78.5%, 71.4%, and 93.0%, respectively. There was a statistical difference in the ORR between the three groups. Further analysis showed that after IC or overall treatment, the CR rate of primary nasopharynx foci in the anti-PD-1 group was significantly higher than the other two groups. The most common adverse effects were hematotoxicity, gastrointestinal toxicity, and transaminase elevation. However, there were no statistical differences in the frequency of any common adverse effects between the three groups.

**Conclusions:**

The addition of anti-PD-1 based on IC significantly improved the short-term efficacy of LA-NPC and toxicities were tolerable.

## Introduction

Nasopharyngeal carcinoma (NPC) is an aggressive epithelial malignancy that arises from the epithelium of the nasopharynx (1). The incidence of NPC is low worldwide, and its distribution has a unique ethnic and geographic distribution pattern, with the highest incidence in southern China and Southeast Asia, especially in the Chinese province of Guangdong (1, 2). Histopathologically, almost all NPC is poorly differentiated or undifferentiated squamous carcinoma, characterized by a high malignant degree, rapid growth, vague symptoms, early cervical lymph node metastasis, and distant metastases (3). Therefore, the majority of NPC patients often appeared with the advanced locoregionally disease at first diagnosis (1–4).

Due to its radiosensitivity, radiotherapy or a combination of radiation and chemotherapy is the mainstay treatment for NPC. For locoregionally advanced nasopharyngeal carcinoma (LA-NPC), the main reasons for the treatment failure of radical radiotherapy (RT) or concurrent chemoradiation (CCRT) are distant metastasis and local recurrence (5, 6). Induction chemotherapy (IC) can decrease the tumor burden, narrow lesions and eliminate distant micro-metastasis over a short period of time, and quickly relieve the symptoms and signs of the compression effect of the tumor on surrounding tissues. Therefore, oncologists have done lots of research on whether the addition of IC to CCRT can further improve the prognosis of patients with LA-NPC. Due to differences in the population studied, sample size, and chemotherapy regimen, previous clinical studies showed that the addition of IC to CCRT has improved the disease-free survival (DFS) but did not significantly improve overall survival (OS) in LA-NPC patients (7–9).

Recently, the final results of several randomized studies on IC have been published, which have demonstrated the prognostic value of IC combined with CCRT in high-risk LA-NPC patients. IC added to CCRT significantly improved DFS and OS, compared with CCRT alone, among patients with LA-NPC (10–12). Following the LA-NPC diagnosis, the standard treatment option is IC, followed by CCRT. The common chemotherapy protocols mainly consist of TPF (docetaxel, cisplatin, and fluorouracil), TPC (aclitaxel, cisplatin, and capecitabine), TP (docetaxel, cisplatin), PF (cisplatin, fluorouracil), GP (gemcitabine, cisplatin) and so on (10–14). These chemotherapy regimens showed similar clinical efficacy and favorable safety profiles in their respective clinical studies.

In addition, NPC is regarded as a highly immune inflammatory tumor because of its unique immune environment, and EGFR is usually highly expressed. Anti-EGFR therapy (Nimotuzumab) plus CCRT in the treatment of LA-NPC (15, 16) and anti-PD-1/PD-L1 immunotherapy combined with chemotherapy (17, 18) in the treatment of refractory for recurrent or metastatic nasopharyngeal carcinoma (RM-NPC) have made significant progress. Currently, clinical investigations on the addition of anti-EGFR or anti-PD-1/PD-L1 immunotherapy to IC are ongoing. Therefore, we conducted this retrospective study to evaluate the short-term efficacy and safety of anti-EGFR or anti-PD-1 immunotherapy with IC for LA-NCP.

## Materials and methods

### Patients

In this study, we retrospectively analyzed the clinical data of NPC patients who received IC at our institution between February 2019 to February 2022. The inclusion criteria were as follows: (1) Age ≥18 years old; (2) Pathologically diagnosed as NPC; (3) LA-NPC including stage III/IVa in accordance with the Eighth Edition of the AJCC staging system, but clinical stage T3/T4N0 was excluded; (4) All patients had a measurable primary lesion and at least one measurable metastatic lymph node according to the Response Evaluation Criteria in Solid Tumors (RECIST) version 1.1. The short-term efficacy evaluation is evaluated by experienced radiation therapists and re-examined by senior physicians; (5) Patients were treated with definitive IC followed by definitive CCRT or RT; (6) Before receiving IC, the patient’s renal function, hepatic function, and hematological parameters were normal. The exclusion criteria included the following: (1) With incomplete clinical data;(2) Have undergone surgery before receiving induction chemotherapy;(3) Patients with apparent immune system diseases, concomitant inflammatory diseases, or blood system diseases; (4) History of severe cardiovascular or pulmonary disease;(5) Patients with a second primary tumor. Written informed consent from the participants was exempted owing to its retrospective design. We have de-identified all patient details such that the identity of any person may not be ascertained in any way.

### Induction chemotherapy

In this study, all patients received two to three cycles of platinum-based IC, including modified TPF (nab-paclitaxel, raltitrexed, and cisplatin), GP (gemcitabine plus cisplatin), and TP (docetaxel/nab-paclitaxel plus cisplatin) regimens. The modified TPF was administered as 260 mg/m^2^ nab-paclitaxel intravenously on day 1, 80 mg/m^2^ cisplatin intravenously on day 1, and 3 mg/m^2^ raltitrexed intravenously on day 1; 2 to 3 cycles were administered at intervals of 3 weeks. GP regimen was administered as gemcitabine (1000 mg/m^2^ on days 1 and 8) and cisplatin (80 mg/m^2^ on day 1) intravenously every 3-week cycle for 2 to 3 cycles. TP consisted of nab-paclitaxel (260 mg/m^2^, d1, intravenous infusion) plus cisplatin (80 mg/m^2^, d1, intravenous infusion) administered every 3 weeks for 2 to 3 cycles. Whether patients received IC combination of PD-1 inhibitors or anti-EGFR depends on the joint decision of the patient, the family, and the attending physician. If the patient received IC combined with anti-PD-1 immunotherapy or anti-EGFR targeted therapy, 200 mg camrelizumab, 240 mg toripalimab, or 200 mg nimotuzumab were given intravenously on the first day of each cycle IC.

### Radiotherapy

All patients received RT or platinum-based CCRT (cisplatin 100mg/m^2^ or lobaplatin 30 mg/m^2^ every three weeks) after the completion of IC, and approximately 50% of these patients continued to receive anti-EGFR (200 mg nimotuzumab, 5-6 cycles) or anti-PD-1 immunotherapy (240 mg toripalimab, 2-3 cycles) during the radiotherapy period. Radiation therapy was delivered with the volumetric modulated arc therapy technique (VMAT). Gross tumor volume (GTV) consisted of the primary tumor and enlarged lymph nodes (GTVnx, the sum of the primary nasopharyngeal site and enlarged retropharyngeal nodes; GTVnd, the clinically involved cervical lymph nodes). High-risk clinical target volume (CTV1) was defined as the area where the metastatic positive lymph nodes are located and the lymphatic drainage area at the next station. The low-risk lymphatic drainage area (CTV2) is defined as the cervical lymphatic drainage area that needs prophylactic irradiation, except CTV1. For the planning target volumes of GTVnx, GTVnd, CTV1, and CTV2, total radiation doses of 69.96 Gy, 69.96 Gy, 60.06 Gy, and 54.45 Gy, respectively, were administered in 33 fractions delivered five times per week, starting on the first day of the first CCRT cycle.

### Therapeutic efficacy and toxicity assessment

The short-term efficacy was assessed at the end of IC and one month after the end of overall treatment by physical examination, nasopharyngeal fiberscope examination, and magnetic resonance imaging (MRI) of the nasopharynx/neck. According to the Response Evaluation Criteria in Solid Tumors version 1.1 (RECIST v1.1), the short-term efficacy of cervical lymph nodes and primary nasopharynx foci was divided into complete remission (CR), partial remission (PR), stable disease (SD), and progressive disease (PD). The overall response (ORR) was defined as the sum of CR and PR, and the disease control rate (DCR) was the sum of CR, PR, and SD.

Other evaluations included routine hematological, biochemistry tests, and physical examinations. Acute hematological and nonhematological toxicities during IC and CCRT/RT were graded according to the Common Terminology Criteria for Adverse Events (CTCAE) v5.0.

### Statistical analysis

Data analysis was performed using SPSS (IBM SPSS 23.0, SPSS Inc). Descriptive statistics were generated for relevant clinical characteristics. Fisher Freeman-Halton test or Pearson Chi-square test was used for the comparison of classified variables. One-way analysis of variance (ANOVA) was used to compare differences in the numerical variables among groups. A p<0.05 was considered statistically significant.

## Results

### Patient characteristics

A total of 206 patients with LA-NPC were included in this study, including 121 (58.7%) patients with IC alone, 57 (27.7%) patients with IC combined with anti-PD-1, and 28 (13.6%) patients with IC combined with anti-EGFR. According to the eighth edition of the AJCC staging system, 66 (32.0%) patients were in stage III and 140 (68.0%) in stage IVA. There was no significant difference in age, gender, T stage, N stage, TNM stage, and pathological characteristics among the IC group, anti-PD-1 group, and anti-EGFR group, but there were differences in specific IC regimens and treatment protocols after IC. Overall, the specific IC regimen was mainly GP (66, 32.1%) alone and GP combined with anti-PD-1 (47, 22.8%), while after IC (84, 41.3%) patients received CCRT and CCRT combined with anti-EGFR (81, 39.3%). The basic clinical characteristics of the LA-NPC patients and the specific regimens are available in **Table 1**.

**Table 1:** Basic clinical characteristics of the patients and the specific regimens.

Characteristic	IC	anti-PD-1	anti-EGFR	χ2/F	*p*-value
No. of the patients (n, %)	121 (58.7)	57 (27.7)	28 (13.6)	/	*/*
Age, years (Median, Range)	46 (18–77)	45 (24–73)	51.5 (20–76)	1.14	0.321
Gender (n, %)				0.398	0.820
Male	90 (74.4)	40 (70.2)	21 (75.0)		
Female	31 (25.6)	17 (29.8)	7 (25.0)		
Clinical stage^&^ (n, %)					
III	44 (36.4)	16 (28.1)	6 (21.4)	2.899	0.235
IVA	77 (63.6)	41 (71.9)	22 (78.6)		
T stage^&^ (n, %)				8.205	0.223
T1	15 (12.4)	10 (17.5)	3 (10.7)		
T2	12 (9.9)	8 (14.0)	0 (0.0)		
T3	53 (43.8)	26 (45.6)	12 (42.9)		
T4	41 (33.9)	13 (22.8)	13 (46.4)		
N stage^&^ (n, %)				5.772	0.217
N1	21 (17.4)	6 (10.5)	5 (17.9)		
N2	56 (46.3)	20 (35.1)	10 (35.7)		
N3	44 (36.4)	31 (54.4)	13 (46.4)		
Histology (nonkeratinizing) (n, %)					
differentiated	17 (14.0)	7 (12.3)	4 (14.3)	0.116	0.943
undifferentiated	104 (86.0)	50 (87.7)	24 (85.7)		
IC regimens (n, %)					
GP	66 (54.5)	47 (82.5)	2 (7.1)	49.395	<0.001*
TP	23 (19.0)	10 (17.5)	14 (50.0)		
TPF	32 (26.4)	0 (0.0)	12 (42.9)		
IC followed by RT regimens (n, %)				54.412	<0.001*
CCRT	67 (55.4)	15 (26.3)	3 (10.7)		
CCRT+anti-EGFR	43 (35.5)	20 (35.1)	18 (64.3)		
CCRT+anti-PD-1	1 (0.8)	14 (24.6)	1 (3.6)		
RT	5 (4.1)	3 (5.3)	0 (0.0)		
RT+anti-EGFR	5 (4.1)	5 (8.8)	6 (21.4)		

SD, standard deviation; RT, radiation therapy; IC, induction chemotherapy; GP, gemcitabine plus cisplatin; TP, docetaxel/nab-paclitaxel plus cisplatin; TPF, nab-paclitaxel, raltitrexed and cisplatin; RT, radical radiotherapy; CCRT, concurrent chemoradiation; ^&^, clinical staging according to the Eighth Edition of the AJCC staging system. Bold indicates the significant values (*p < 0.05).

### Short-term efficacy after IC for primary nasopharynx lesions

At the end of induction chemotherapy and one month after the end of overall treatment, the efficacy of primary nasopharynx lesions and cervical lymph node metastasis was evaluated according to the Response Evaluation Criteria in Solid Tumors version 1.1 (RECIST v1.1).

After induction chemotherapy, the clinical efficacy of primary nasopharynx lesions was evaluated: the response in the IC alone group was CR in 0.8%, PR in 67.8%, and SD in 31.4%; for anti-PD-1 group CR in 14%, PR in 80.7% and SD in 5.3%; for anti-EGFR group CR in 0%, PR in 67.9% and SD in 31.2%; while no patient showed disease progression in the primary site.

The ORR rate of IC, anti-EGFR and anti-PD-1 groups were 68.60% (83/121), 67.9%(19/28), and 94.7% (54/57), respectively. The ORR was statistically different between the three groups (χ 2 = 17.30 p < 0.001). There was no significant difference between IC and anti-EGFR group (χ 2 = 0.006, *p*=0.94), but there was significant difference between IC and anti-PD-1 group (χ 2 = 14.936, *p* < 0.001), and between anti-PD-1 and anti-EGFR group (Fisher Freeman-Halton test: *p* = 0.002). Further information is given in **Table 2**.

**Table 2:** Short-term efficacy after induction chemotherapy.

Lesion site	Group	No.	CR	PR	SD	PD	ORR	χ2 test	
		(n)	(n, %)	(n, %)	(n, %)	(n, %)	(n, %)	χ2	*p*
Nasopharynx								15.49	<0.001
	anti-EGFR	28	0 (0%)	19 (67.9%)	9 (31.2%)	0 (0%)	19 (67.9%)		
	anti-PD-1	57	8 (14%)	46 (80.7%)	3 (5.3%)	0 (0%)	54 (94.7%)		
	IC	121	1(0.8%)	82 (67.8%)	38 (31.4%)	0 (0%)	83 (68.6%)^*&^		
Lymph node								7.60	0.021
	anti-EGFR	28	8(28.6%)	12 (42.9%)	8 (28.6%)	0 (0%)	20 (71.4%)		
	anti-PD-1	57	21(36.8%)	32 (56.1%)	4 (7.0%)	0 (0%)	53 (93.0%)		
	IC	121	27(22.3%)	68 (56.2%)	26(21.5%)	0 (0%)	95 (78.5%)^*&^		

IC, induction chemotherapy; CR, complete remission; PR, partial remission; SD, stable disease; PD, progressive disease; ORR, the sum of CR and PR; Note: CR + PR were considered effective; SD + PD were considered invalid. Bold indicates the significant values (p < 0.05). *compared with the anti-EGFR group, p < 0.017; ^&^ compared with the anti-PD-1 group, p <0.017.

### Short-term efficacy after IC for cervical lymph nodes

The short-term efficacy of cervical lymph nodes was evaluated: the response in the IC alone group was CR at 22.3%, PR at 56.2%, and SD at 21.5%; for the anti-PD-1 group CR at 36.8%, PR at 56.1%, and SD in 7.0%; for anti-EGFR group CR in 28.6%, PR in 42.9% and SD in 8.0%. No patients progressed by the end of treatment.

The ORR rate of IC, anti-EGFR, and anti-PD-1 was 78.5% (95/121), 93.0% (53/57), and 71.4% (20/28), respectively. The ORR was statistically different between the three groups (χ 2 = 7.60, *p* = 0.021). Regarding the ORR of cervical lymph nodes, significant differences were observed between anti-PD-1 and IC group χ 2 = 5.789, *p* = 0.016), and between anti-PD-1 and anti-EGFR group (Fisher Freeman-Halton test: *p* = 0.016); however, no significant difference was observed between IC and anti-EGFR group (χ 2 = 0.648, *p* = 0.421) **Table 2**.

### Subgroup analysis of short-term efficacy in GP and GP + anti-PD-1

In this retrospective study, GP was the main chemotherapy regimen, so we compared the short-term efficacy between the GP and the GP + anti-PD-1 group by case sample size. The ORR rate of primary nasopharynx lesions in GP group and GP + anti-PD-1 group was 72.7% vs 95.7%, which was statistically significant (χ 2 = 9.984, *p* = 0.002); while the ORR rate in cervical lymph nodes was 86.4% vs 91.5%, which had no statistical difference (χ 2 = 0.708, *p* = 0.400) **Table 3**.

**Table 3:** Comparison of short-term efficacy between GP and GP + anti-PD-1 group.

Lesion site	Group	No.	CR	PR	SD	PD	ORR	χ2 test	
		(n)	(n, %)	(n, %)	(n, %)	(n, %)	(n, %)	χ2	*P*
Nasopharynx								9.984	0.002
	GP+ anti-PD-1	47	8 (17.0%)	37 (78.7%)	2 (4.3%)	0 (0%)	45 (95.7%)		
	GP	66	1 (1.5%)	47 (71.2%)	18 (27.3%)	0 (0%)	48 (72.7%)		
Lymph node								0.708	0.400
	GP+ anti-PD-1	47	15 (31.9%)	28 (59.6%)	4 (8.5%)	0 (0%)	43 (91.5%)		
	GP	66	18 (27.3%)	39 (59.1%)	9 (13.6%)	0 (0%)	57 (86.4%)		

IC, induction chemotherapy; CR, complete remission; PR, partial remission; SD, stable disease; PD, progressive disease; ORR, the sum of CR and PR; Note: CR + PR were considered effective; SD + PD were considered invalid; GP, gemcitabine plus cisplatin; Bold indicates the significant values (p < 0.05).

### Short-term efficacy of the different IC regimens

We made a subgroup analysis of the short-term efficacy of 121 patients treated with induction chemotherapy alone. The ORR rates of GP, TP, and TPF to the primary nasopharynx lesions were 72.7%, 56.5%, and 68.8%, respectively. The three groups had no statistical difference (χ 2 = 2.080, *p* = 0.353). At the same time, there was no statistical difference between GP and TP (χ 2 = 2.077, *p* = 0.150), GP and TPF (χ 2 = 3.017, *p* = 0.082), TP and TPF (χ 2 = 0.278, *p* = 0.598).

The ORR rates of GP, TP, and TPF to the cervical lymph nodes were 86.4%, 65.2%, and 71.9%, respectively. There was no statistical difference among the three groups (χ 2 = 5.657, *p* = 0.059). At the same time, there was no statistical difference between GP and TP (Fisher Freeman-Halton test, *p* = 0.035 > 0.017), GP and TPF (χ 2 = 3.017, *p* = 0.082), TP and TPF (χ 2 = 0.278, *p* = 0.598) **Table 4**.

**Table 4:** Subgroup analysis by the specific chemotherapy regimens.

Lesion site	IC Group	No.	CR	PR	SD	PD	ORR	χ2 test	
		(n)	(n, %)	(n, %)	(n, %)	(n, %)	(n, %)	χ2	*p*
Nasopharynx								2.080	0.353
	GP	66	1 (1.5%)	47 (71.2%)	18 (27.3%)	0 (0%)	48 (72.7%)		
	TP	23	0 (0.0%)	13 (56.5%)	10 (43.5%)	0 (0%)	13 (56.5%)		
	TPF	32	0 (0.0%)	22 (68.8%)	10 (31.3%)	0 (0%)	22 (68.8%)^*&^		
Lymph node								5.657	0.059
	GP	66	18 (27.3%)	39 (59.1%)	9 (13.6%)	0 (0%)	57 (86.4%)		
	TP	23	2 (8.7%)	13 (56.5%)	8 (34.8%)	0 (0%)	15 (65.2%)		
	TPF	32	7 (21.9%)	16 (50.0%)	9 (28.1%)	0 (0%)	23 (71.9%)^*&^		

IC, induction chemotherapy; CR, complete remission; PR, partial remission; SD, stable disease; PD, progressive disease; ORR, the sum of CR and PR; Note: CR + PR were considered effective; SD + PD were considered invalid; GP, gemcitabine plus cisplatin; TP, docetaxel/nab-paclitaxel plus cisplatin; TPF, nab-paclitaxel, raltitrexed and cisplatin; Bold indicates the significant values (p < 0.05). *compared with the TP group, p < 0.017; ^&^ compared with the TPF group, p<0.017.

### Short-term efficacy after overall treatment

After overall treatment, the ORR rate of nasopharyngeal lesions and cervical lymph nodes in all patients was close to 100%, only three patients who only received induction chemotherapy before radiotherapy were evaluated as SD (2 nasopharyngeal lesions and one cervical lymph node).

Further analysis found that after overall treatment, the CR rate of nasopharyngeal lesions in the anti-PD-1 group was significantly higher than the IC or anti-EGFR group (84.2% vs. 52.9% vs. 53.6%, χ 2 = 18.240, *p* <0.001). There was no significant difference between IC and anti-EGFR group (Fisher Freeman-Halton test, χ 2 = 0.228, *p*=1.0), but there was significant difference between IC and anti-PD-1 group (Fisher Freeman-Halton test, χ 2 = 16.817, *p* < 0.001), and between anti-PD-1 and anti-EGFR group (χ 2 = 9.188, *p* = 0.002). The short-term efficacy of cervical lymph nodes did not differ significantly between the three groups (Fisher Freeman-Halton test, χ 2 = 5.358, *p <*0.217) **Table 5**.

**Table 5:** Short-term efficacy after overall treatment.

Lesion site	Group	No.	CR	PR	SD	PD	F^#^ test	
		(n)	(n, %)	(n, %)	(n, %)	(n, %)	χ2	*p*
Nasopharynx							18.240	<0.001
	anti-EGFR	28	15 (53.6%)	13 (46.4%)	0 (0%)	0 (0%)		
	anti-PD-1	57	48 (84.2%)	46 (15.8%)	0 (0%)	0 (0%)		
	IC	121	64 (52.9%)	55 (45.5%)	2 (1.7%)	0 (0%)^*&^		
Lymph node							5.358	0.217
	anti-EGFR	28	20(71.4%)	8 (28.6%)	0 (0%)	0 (0%)		
	anti-PD-1	57	50(87.7%)	7 (12.3%)	0 (0%)	0 (0%)		
	IC	121	92(76.0%)	28 (23.1%)	1 (0.8%)	0 (0%)		

IC, induction chemotherapy; CR, complete remission; PR, partial remission; SD, stable disease; PD, progressive disease; F^#^, Fisher Freeman-Halton test. Bold indicates the significant values (p < 0.05). *compared with the anti-EGFR group, p < 0.017; ^&^ compared with the anti-PD-1 group, p<0.017.

### Toxicity

Acute toxicity during induction chemotherapy was assessed between the three groups, and no severe adverse reactions occurred, as shown in **Table 6**. The most common adverse effects were hematotoxicity, gastrointestinal toxicity, and the elevation of transaminase. However, there were no statistical differences in the frequency of any common adverse effects between the three groups (p>0.05).

**Table 6:** Major adverse events during induction chemotherapy.

Adverse event	IC	anti-PD-1	anti-EGFR	χ2/F*	
				χ2	*p*
Leukopenia				5.044	0.283
G0 + 1	71 (58.7%)	26 (45.6%)	18 (64.3%)		
G2	35 (28.9%)	19 (33.3%)	8 (28.6%)		
G3 + 4	15 (12.4%)	12 (21.1%)	2 (7.1%)		
Neutropenia				4.280	0.369
G0 + 1	62 (51.2%)	25 (43.9%)	16 (57.1%)		
G2	34 (28.1%)	13 (22.8%)	7 (25.0%)		
G3 + 4	25 (20.7%)	19 (33.3%)	5 (17.9%)		
Hemoglobin				8.467	0.051
G0 + 1	107 (88.4%)	43 (75.4%)	27 (96.4%)		
G2	11 (9.1%)	13 (22.8%)	1 (3.6%)		
G3 + 4	3 (2.5%)	1 (1.8%)	0 (0%)		
Thrombocytopenia				4.342	0.325
G0 + 1	108 (89.3%)	48 (84.2%)	28 (100%)		
G2	7 (5.8%)	4 (7.0%)	0 (0%)		
G3 + 4	6 (5.0%)	5 (8.8%)	0 (0%)		
ALT/AST elevated				3.522	0.172
G0 + 1	117 (96.7%)	52 (91.2%)	25 (89.3%)		
G2 + 3	4 (3.3%)	5 (8.8%)	3 (10.7%)		
Nausea				0.200	0.905
G0 + 1	106 (89.1%)	52 (91.2%)	25 (89.3%)		
G2	13 (10.9%)	5 (8.8%)	3 (10.7%)		
Vomiting				3.911	0.102
G0 + 1	114 (94.2%)	57 (100%)	28 (100%)		
G2	7 (5.8%)	0 (0%)	0 (0%)		

IC, induction chemotherapy; ALT, alanine aminotransferase; AST, aspartate transaminase; χ2/F*, Chi-square test or Fisher Freeman-Halton test.

## Discussion

Currently, the multimodal treatment approach, combining radiotherapy and chemotherapy, is the primary treatment strategy in locoregionally advanced nasopharyngeal carcinoma (LA-NPC); up to 10–20% of LA-NPC patients still develop local or metastatic relapse and die from this disease (19, 20). Induction chemotherapy (IC) can decrease the tumor burden, narrow lesions, eliminate distant micro-metastasis over a short period, and quickly relieve the symptoms and signs of the compression effect of the tumor on surrounding tissues. Therefore, oncologists have done lots of research on whether adding IC to concurrent chemoradiation (CCRT) can further improve the prognosis of patients with LA-NPC. The final overall survival (OS) analysis of a multicenter, randomized phase III trial showed that gemcitabine and cisplatin induction chemotherapy before concurrent chemoradiotherapy (CCRT) significantly improved OS in patients with LA-NPC without increasing the risk of late toxicities due to cancer therapies (12). Furthermore, It is particularly noteworthy that the depth of the tumor response to IC for LA-NCP significantly and positively correlates with OS, the 5-year OS of patients with complete response (CR), partial response (PR), and stable/progressive disease (SD/PD) was 100%, 88.4%, and 61.5%, respectively (*p*<0.05). Therefore, we conducted this retrospective study to evaluate the short-term efficacy and safety of anti-EGFR targeted therapy or anti-PD-1 immunotherapy with IC for LA-NCP. In this study, we found that the addition of anti-PD-1 immunotherapy in induction chemotherapy increased the short-term efficacy (anti-PD-1 vs IC vs anti-EGFR, the ORR of primary nasopharynx lesions/cervical lymph nodes, 94.7%/93.0% vs 68.6%/78.5% vs 67.9%/71.4%, p <0.001/p=0.021), especially significantly increased the CR rate of primary nasopharynx lesions, and did not increase the treatment-related side effects.

Epstein-Barr virus (EBV) infection has been consistently identified as an essential risk factor for the progression of nasopharyngeal carcinoma (NPC) (21). Chronic EBV infection, numerous lymphocyte infiltrates, high PD-L1 expression, and several key immune molecules involved in T cell activation can be frequently observed in EBV-induced NPC tumor tissues (22). In addition to having very potent antigens, NPC has an elevated level of interferon response and is associated with a higher proportion of antigen-presenting cells (23). Based on the particular immune landscape of NPC, NPC is considered a highly immuno-inflammatory disease, which makes patients more suitable for immunotherapy.

In recent years, the blockade of inhibitory immune checkpoints programmed death-1/programmed death ligand-1 (PD-1/PD-L1) has made a breakthrough treatment advance. The efficacy and safety of many PD-1/PD-L1 inhibitors have previously been demonstrated in patients with refractory for recurrent or metastatic nasopharyngeal carcinoma (RM-NPC). One of the studies was on the efficacy and safety of camrelizumab in RM-NPC. The results of the phase I trials showed that 34% (95% CI 24-44) of evaluable patients had an overall response with a median follow-up of 9.9 months (24). The combination of camrelizumab and GP regimen showed promising anti-tumor activity and manageable safety. Subsequently, randomized phase III trials explored the addition of camrelizumab to the GP regimen as the first-line treatment for RM-NPC. 263 eligible patients were randomly assigned to the camrelizumab or the placebo group. Results of the evaluation showed that the progression-free survival (PFS) was significantly prolonged in the carellizumab group (9.7months vs. 6.9months, *p*=0·0002), and the most common grade 3 or above toxicities were hematotoxicity in both groups (18). Another multicenter randomized phase III trial included 289 patients with RM-NPC, mainly evaluating the efficacy and safety of the GP regimen combined with toripalimab or placebo in the first-line treatment. The prespecified interim PFS analysis showed that the PFS in the toripalimab group significantly improved compared to the placebo group (11.7 versus 8.0 months, HR = 0.52 (95%CI: 0.36-0.74), p = 0.0003). The grade 3 or higher adverse events were similar between the two groups (89.0 versus 89.5%). Based on these two studies, toripalimab and camrelizumab were approved for the treatment paradigms of RM-NPC in China.

Based on the preclinical evidence and promising results of anti-PD-1/PDL-1 immune checkpoint inhibitors in RM-NPC, the addition of immunotherapy in the current combined treatment modalities for LA-NPC is a hot clinical research topic, and many prospective phase II or III trials are ongoing (25). Ongoing studies mainly focus on immunotherapy’s role in locally recurrent NPC. For LA-NPC at initial diagnosis, the phase III clinical trials on adding camrelizumab, toripalimab, or sintilimab to IC and CCRT are currently in the participant recruitment phase (NCT04453826, NCT03700476, NCT04557020, NCT04557020) (25).

Since August 2020, our hospital has been trying to add camrelizumab or toripalimab to IC for newly diagnosed LA-NPC, so we conducted this retrospective analysis. Our retrospective data showed that the addition of anti-PD-1 immunotherapy to IC has a remarkable short-term efficacy, which is significantly better than that of patients with simple IC or IC combined with targeted therapy (anti-EGFR), and the frequency of any of the common adverse effects between the three groups are similar (*p* >0.05). For primary nasopharynx lesions, the ORR rate of IC, anti-EGFR and anti-PD-1 group were 68.60% (83/121), 67.9%(19/28)and 94.7% (54/57), respectively (χ 2 = 17.30 *p* < 0.001). The short-term efficacy of cervical lymph nodes also was evaluated: the ORR rate of IC, anti-EGFR and anti-PD-1 group were 78.5% (95/121), 93.0%(53/57)and 71.4% (20/28), respectively (χ 2 = 7.60, *p* = 0.021). After overall treatment, the ORR rate of nasopharyngeal lesions and cervical lymph nodes in all patients was close to 100%. Only three patients who received IC before radiotherapy were evaluated as SD (2 nasopharyngeal lesions and one cervical lymph node). Further analysis found that the CR rate of nasopharyngeal lesions in the anti-PD-1 group was significantly higher than the IC or anti-EGFR group (84.2% vs. 52.9% vs. 53.6%, χ 2 = 18.240, *p* <0.001). However, because the specific induction chemotherapy regimen was not uniform in our retrospective study, we conducted a subgroup analysis of the GP regimen combined with anti-PD-1 immunotherapy and GP regimen according to the sample size of patients. However, because the specific induction chemotherapy regimen was not uniform in our retrospective study, we conducted a subgroup analysis of the GP regimen combined with anti-PD-1 immunotherapy and GP regimen according to the sample size of patients. Subgroup analysis revealed that the ORR rate of primary nasopharynx lesions in the GP group and GP + anti-PD-1 group was 72.7% vs. 95.7%, which was statistically significant (χ 2 = 9.984, *p* = 0.002). In particular, the CR rate of primary nasopharynx lesions in the GP + anti-PD-1 group was significantly higher than in the GP group, which were 17.0% and 1.5%, respectively.

In addition, previous studies have demonstrated that epidermal growth factor receptor (EGFR) is closely related to the malignant transformation of squamous cells and the proliferation of tumor cells. Molecular-targeted therapies using EGFR as the target is widely used in the clinical treatment of head and neck tumors. It is known that the levels of EGFR are overexpressed in more than 70% of patients with NPC and are closely associated with poor prognosis (26, 27). Nimotuzumab is the first monoclonal antibody authorized in China for EGFR-targeted therapy. The combination of CCRT with nimotuzumab has shown an excellent efficacy and safety profile in the treatment of NPC. A retrospective study of 730 patients with stage III-IVb NPC showed that the 5-year OS rate in the CCRT plus nimotuzumab group was significantly higher than that in the CCRT group (88.91% versus 78.30%, *p* = 0.006). The preliminary results of a recent phase III clinical study came from the 2022 ASCO annual meeting, which included 482 patients with locally advanced NPC. The results showed that the OS in the nimotuzumab combined with the CCRT group were significantly higher than those in the control group (76.9% versus 64.3%, log-rank = 4.125, p = 0.042), and the adverse effects were similar in both groups (15). However, there is currently no high-quality study on adding nimotuzumab to induction chemotherapy in the treatment of LA-NPC. Our study showed that adding nimotuzumab to IC did not significantly increase the ORR rate of patients. This might be attributed to the short follow-up period and the insufficient number of cases. Furthermore, the clinical practice of LA-NPC patients involves the application of various IC regimens, such as TPF, TP, PF, and GP, all of these chemotherapy regimens showed similar clinical efficacy and favorable safety profiles in their respective clinical studies (10–14). We made a subgroup analysis of the short-term efficacy of 121 patients treated with induction chemotherapy alone. There was no significant difference in ORR rates of cervical lymph nodes and primary nasopharynx lesions between the GP, TP, and TPF groups.

Nevertheless, we acknowledge that several limitations exist concerning this study. First, this is a retrospective study with a small number of patients, especially only 28 patients who received IC combined with targeted therapy, which may have resulted in potential selection biases. Furthermore, the IC regimens in this study were not unified, although our subgroup analysis showed no significant among-protocol difference in short-term efficacy. Finally, due to the short follow-up time in this study, only the short-term efficacy was assessed, but no long-term efficacy indicators such as OS or DFS were measured. Despite the above limitations, this study emphasizes that combining anti-PD-1 immunotherapy and chemotherapeutic drugs in IC regimens can significantly enhance the short-term efficacy of patients with LA-NPC.

## Conclusions

In conclusion, our results showed that the addition of anti-PD-1 immunotherapy on the basis of induction chemotherapy significantly improved the short-term efficacy of locoregionally advanced nasopharyngeal carcinoma and toxicities were tolerable. However, its long-term efficacy should be confirmed by further follow-up and phase III randomized clinical trials.

## Data availability statement

The raw data supporting the conclusions of this article will be made available by the authors, without undue reservation.

## Ethics statement

The studies involving human participants were reviewed and approved by the institutional ethics committees of Cancer Hospital, Chinese Academy of Medical Sciences. Written informed consent for participation was not required for this study in accordance with the national legislation and the institutional requirements.

## Author contributions

JJ and YZ were responsible for the primary concept and the design of the study. XX and PC performed the data capture and analysis. XX drafted the manuscript. All authors contributed to the article and approved the submitted version.
